# Effect of Psychophysiological Stress and Socio-Emotional Competencies on the Clinical Performance of Nursing Students during a Simulation Practice

**DOI:** 10.3390/ijerph18105448

**Published:** 2021-05-19

**Authors:** Elba Mauriz, Sandra Caloca-Amber, Lucía Córdoba-Murga, Ana María Vázquez-Casares

**Affiliations:** 1 Department of Nursing and Physiotherapy, Universidad de León, Campus de Vegazana, 24071 León, Spain; scaloa00@estudiantes.unileon.es (S.C.-A.); lcordm01@estudiantes.unileon.es (L.C.-M.); ana.vazquez@unileon.es (A.M.V.-C.); 2 Institute of Food Science and Technology (ICTAL), La Serna 58, 24007 León, Spain

**Keywords:** psychophysiological response, socio-emotional competencies, stress, anxiety, cognitive load, self-efficacy, clinical simulation

## Abstract

Psychophysiological stress can affect the cognitive response and effective learning of students during medical simulation practices. This study aimed to explore the effect of psychophysiological stress and socio-emotional competencies on clinical performance during a simulation experience. A pre-test/post-test design was used to assess physiological (blood pressure, heart rate and blood oxygen saturation) and psychological parameters (stress and anxiety) as well as socio-emotional skills (cognitive load, self-efficacy and motivation) in nursing students (*n* = 40) before and after the simulation of a cardiopulmonary resuscitation practice. Physiological responses showed statistically significant differences between pre-test and post-test conditions for blood pressure and heart rate (*p* < 0.0001). Moderate and significant correlations were also observed when comparing self-efficacy with stress (*r =* −0.445, *p* = 0.004), anxiety (*r =* −0.467, *p* = 0.002) and motivation (*r =* −0.406, *p* = 0.009) measures. Similarly, cognitive-load dimensions were significantly associated with either physiological (*r =* −0.335, *p* = 0.034) or psychological (*r =* −0.448, *p* = 0.004) indicators. The analysis of multiple regression models revealed a relationship between the effectiveness of the simulated experience, post-test blood oxygen saturation, heart rate, workload and self-efficacy (R^2^ = 0.490; F (3, 39) = 8.305; *p* < 0.0001; *d* = 1.663). Therefore, the evaluation of psychophysiological parameters and socio-emotional skills seems to provide a promising framework for predicting the quality of simulated clinical practices.

## 1. Introduction

The incorporation of instructional methods in a didactic environment is essential to improve the efficacy of the learning process. From this perspective, simulation is an educational methodology ideally suited to acquiring experience in low-risk training environments [1,2,3,4]. In particular, medical simulation enables immersion in interactive scenarios that reproduces essential aspects of the real world [2,5]. Learning in highly realistic environments prepares students to face clinical situations that require direct care while ensuring patient safety [6]. Moreover, the amplification of real experiences in safe and controlled environments provides a solid framework for the development of critical thinking, self-confidence and clinical judgment [3,4,7,8,9].

Despite the intrinsic value of simulation education, a lack of experience and emotional control may trigger a stress response that can interfere with student performance [6,10,11]. The anticipation of critical situations and the perception of being observed induces the activation of the autonomic nervous system (ANS) and the hypothalamic–pituitary–adrenal (HPA) axis associated with superior cortical functions [6,11,12]. The sympathetic response of the autonomous system leads to the increase of blood pressure, heart rate, skin temperature and anaerobic metabolism, whereas the HPA activation is associated with the secretion of cortisol and diminished blood flow in the cortical system [10,11,13]. The complementary effect of the ANS and HPA axis leads to an adaptive response that contributes to confronting threats, risks and uncertainties through the mobilization of energy and BP maintenance [6,12]. However, acute exposure to stress and anxiety during a simulation practice can negatively affect the acquisition of knowledge, skills, and attitudes [6,14]. The generation of elevated stress has been associated with the alteration of cognition and memory [15,16]. Similarly, the anxiety experienced as a consequence of autonomous system activation can undermine cognitive resources and affect the process of managing information [17,18].

In this sense, the mental effort associated with decision making, namely cognitive load, is closely related to the expertise and emotional state of the learner for the successful completion of a specific task [17,19]. Self-efficacy, or sense of confidence in the ability to perform the task, also plays an important role in achieving optimal performance [20,21,22]. These socio-emotional skills can compromise the quality of clinical practice and determine the effectiveness of the simulation-based learning experience. Thus, the extent to which stress and anxiety levels can affect simulation outcomes not only relies on the stressors of the learning environment but also on the socio-emotional competencies of the participants [16,17,23].

Although several investigations have examined the impact of the physiological response on either clinical and non-clinical context [6,10,24], the combined effect of psychophysiological stress and socio-emotional competencies on the training performance has not been thoroughly addressed [15,16]. For instance, the assessment of the psychophysiological response has been measured in simulated critical situations such as military training [10,25] or high fidelity and real-life emergency scenarios [26,27,28]. The effect of physical and biochemical stress markers on learning has been evaluated by monitoring variations in vital signs, anxiety state, electrodermal activity and salivary, cortisol or lactate levels [11,24,26,29,30].

On the other hand, the influence of socio-emotional skills on clinical performance has been examined in a variety of simulated environments [31,32]. Cognitive load and self-confidence have been associated with anxiety levels in healthcare providers and nursing students during clinical simulations [17,33]. Self-efficacy and critical thinking have also been used to compare the effectiveness of simulation education regarding traditional clinical models [3,4]. Likewise, the analysis of socio-emotional competencies has been devoted to predicting the academic and clinical performance of nursing students in a simulated practice [31].

However, the concurrent impact of physiological, psychological and socio-emotional variables on performance outcomes has been poorly explored and quantified in simulated clinical scenarios. To address this gap, this study was designed to evaluate the effect of psychophysiological stress and socio-emotional competencies on the performance of nursing students undertaking a simulated cardiopulmonary resuscitation practice. Since both factors can impair the acquisition of technical and non-technical skills, we hypothesized that clinical performance may be associated with physiological and psychological markers of stress as well as with cognitive load, motivation and self-efficacy measurements.

## 2. Materials and Methods

### 2.1. Study Design and Subjects

This study was a continuation of a quasi-experimental study to evaluate the effect of facial skin temperature on the perception of anxiety in nursing students [34]. A convenience sample of 40 participants was recruited among nursing students in their second year of a Bachelor of Science (*n* = 21) and postgraduate registered nurses of a Master of Science in Advanced Clinical Nursing (*n* = 19) during the 2018–2019 academic year. Both groups had received specific training in both Basic Life Support and medium-fidelity simulation during their academic training.

Students participated voluntarily and were informed of the study subject through informed consent, which guaranteed their anonymity and confidentiality, and that a decision to withdraw from the study would not affect their academic progress. Participation was not part of the mandatory program and did not include remuneration or course credit. The recruitment process was carried out via meetings at lecture time and online postings. There were no exclusion criteria other than having to be enrolled in a corresponding academic program. Ethical approval was granted by the University’s Ethics Committee following the Helsinki Declaration guidelines.

### 2.2. Study Protocol

**Figure 1:**
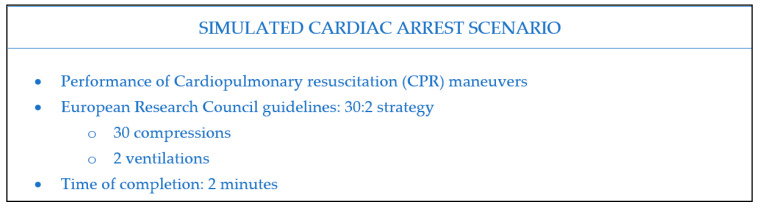
Schematic representation of the simulated cardiac arrest scenario.

The study was performed in two different classrooms. Physiological measurements and self-administered questionnaires were obtained in a training classroom while practical skills were evaluated in the simulation classroom. A simulated cardiac arrest scenario was used to monitor the performance of cardiopulmonary resuscitation (CPR) maneuvers. The scheme of the simulation procedure is presented in Figure 1. Participants were asked to perform CPR individually without receiving feedback of any type. The same protocol was applied to each group on different days between January and February of 2019.

### 2.3. Data Collection

#### 2.3.1. Sociodemographic Data

Personal information including sex, age, graduation status, previous work experience in special services, and basic and advanced life support was obtained from each participant by a questionnaire.

#### 2.3.2. Vital Signs

Measurements of physiological parameters were obtained in triplicate prior to (pre-test) or after (post-test) the simulation practice. The average of 3 measurements was used as a standard value. Systolic blood pressure, diastolic blood pressure and heart rate were measured by an HEM-FL31 (OMRON Healthcare Europe B.V., Hoofddorp, The Netherlands) validated digital blood-pressure monitor, and partial oxygen saturation by pulse oximetry using the pulse oximeter MD300C12 (Beijing Choice Electronic Technology Co., Ltd., Beijing, China).

#### 2.3.3. Technical Skills

The assessment of CPR outcomes was carried out by Little Anne QCPR^®^ (Laerdal Medical^®^, Stavanger, Norway) medium-fidelity simulation mannequin and the QRCP Learner version 1.15.11 mobile app programmed to evaluate a situation of cardiac arrest in asystole [35].

The mannequin, synchronized with the QCPR application, allowed the analysis of the quality of the CPR. Performance scores were obtained from data involving ventilation (overall ventilation score, total number of ventilations (%), ventilations with adequate and excessive chest elevation (%)) and compressions (number of compressions, compressions of adequate depth (%), compressions with total re-expansion of the thorax (%), adequate compression ratio (%), average in mm of compression depth, average compression rate per minute, flow fraction (% of continuous compression time)). Using a mathematical formula, the application calculated the total score on the effectiveness of the resuscitation in a range of 0 to 100 expressed as a percentage (%).

#### 2.3.4. Psychological Stress and Socio-Emotional Competencies

The most common socio-emotional competencies among nursing students (anxiety, stress, workload, self-efficacy and motivation) were evaluated through the following instruments.

- State-Trait Anxiety Inventory (STAI): This questionnaire is considered one of the most effective tools for measuring anxiety. It consists of two subscales, each composed of 20 items that evaluate two different concepts: anxiety as a state (emotional condition at a given time) and anxiety as a trait (anxious propensity of the person in a stable way). The total scale score ranges from 0 to 60 points. The Spanish questionnaire was validated with a Cronbach’s alpha of 0.94 for the anxiety state subscale [36]. In this study, the state subscale was self-rated by the participants before and after the simulation. The response system consists of a Likert scale from 0 to 3; 0 (nothing), 1 (something), 2 (enough) and 3 (much). Higher scores indicate a higher level of self-perceived anxiety.
- Visual analog scale of stress (VAS): This scale consists of a 100 mm line with equidistant points between 0 and 10 from 1 (very little) to 10 (very much), where participants indicate the intensity of the stress at that time. VAS scores were recorded immediately before and after the completion of the CPR performance.
- NASA Task Load Index (NASA TLX): This multidimensional assessment instrument provides a global workload score in a given task, based on a weighted average of the scores at six subscales that refers to mental, physical and temporary demands as well as performance, effort and frustration. The scale consists of two phases: the first is performed before the task, in this case, before the simulation (weighting phase), and the other, comes immediately after performing the task (scoring phase). This scale has been used in several studies to assess the mental load of the task and its variation in the presence of a stressful situation in high-fidelity simulation environments [37]. The internal consistency of NASA TLX for a sample of 398 Spanish workers demonstrated a Cronbach alpha coefficient α.69 [38]. Participants completed the scale before the simulation practice.
- Situational motivation scale (SIMS): The SIMS consists of 16 statements that evaluate intrinsic motivation (items 1, 5, 9, and 13), identify regulation (items 2, 6, 10, and 14) and external regulation (items 3, 7, 11, and 15), and demotivation (items 4, 8, 12 and 16), according to Deci and Ryan’s Theory of Self-Determination, 1985. Each of the statements of the scale answers the question: “Why are you involved in this task/activity at this time?” Then, through a Likert-type scale, it is rated from 1 to 7 on how much each statement corresponds to its perception of the activity performed [21,39]. The SIMS scale has high internal consistency, with Cronbach’s alpha coefficient ranging between 0.77 and 0.87 [39]. The motivation was recorded before the simulation.
- Basic cardiopulmonary resuscitation (CPR) self-efficacy scale: A baseline measure of self-efficacy was obtained before CPR performance. This scale was designed and validated in Spanish in a sample of 1400 health professionals who presented high reliability (Cronbach’s alpha > 0.92). The scale consisted of 8 items valued on a Likert scale from 1 to 6, with 1 being the lowest confidence value and 6 the highest confidence value in the task performed [10,26].

### 2.4. Data Analysis

Continuous variables were expressed as mean values ± standard deviation (SD). The Kolmogorov–Smirnov test was applied to determine data normality. Differences between student groups were analyzed with independent *t*-tests. A comparison between pre-test and post-test simulation scores was completed using Student’s t-test paired and a Wilcoxon-signed rank test for normal and non-normal distribution variables, respectively. Bivariate correlations were used to assess associations between physiological, psychological, socio-emotional variables and CPR quality parameters. Testing was conducted on several multiple linear-regression models in which CPR effectiveness, workload dimensions or self-efficacy scores were considered as dependent variables with the rest of the variables as predictors. The software package SPSS for Windows version v.26 (IBM SPSS, Inc., Chicago, IL, USA) was used for data analysis. A *p*-value of < 0.05 was set as Statistical for all analyses.

## 3. Results

A total of 40 students completed the study (34:6 female–male). Participants were within the 19–36 age range (mean age 22.35 ± 3.39). Differences in sociodemographic characteristics between undergraduate (*n* = 21) and postgraduate (*n* = 19) students were not statistically significant. Similarly, no significant differences were observed in the rest of the studied variables except for the evaluation of performance (CPR global effectiveness) (*p* = 0.027) and the workload estimation (*p* = 0.032).

### 3.1. Vital signs and Stress Variables

Systolic blood pressure (mm Hg), diastolic blood pressure (mm Hg) and heart rate (beats/min) significantly increased from pre-test to post-test simulation measurements (*p* < 0.0001) (Table 1). STAI and VAS post-simulation scores also showed moderate increases over pre-simulation results. Oxygen blood saturation did not increase at the end of the simulation (*p* = 0.831).

### 3.2. Socio-Emotional Competencies

Mean scores obtained in the socio-emotional scales presented high values for the self-efficacy scale (34.08 ± 6.37), and most of the dimensions of the NASA TLX workload questionnaire (64.67 ± 14.51). Medium values were observed for the dimensions of the SIMS motivation scale related to intrinsic motivation (21.58 ± 4.314) and identified regulation (23.23 ± 4.086), while lower scores were obtained for external regulation (12.48 ± 5.809) and demotivation (5.65 ± 3.431) (Table 2).

### 3.3. Vital Signs and Socio-Emotional Competencies versus Performance

Correlation analyses were performed to assess the relationship among variables. First, the analysis of physiological parameters revealed positive and significant associations between the pre-test heart-rate and blood-pressure values (*r =* 0.448 (systolic): *r* = 0.652 (diastolic), *p* < 0.0001), before and after the simulation procedure. On the other hand, a negative relationship was observed between pre-test blood oxygen saturation and systolic blood pressure (*r* = −0.330, *p* < 0.038), regardless of the moment of analysis.

As for psychological variables, a statistically significant and positive association was observed between post-test STAI scores and post-test blood pressure (systolic and diastolic), whereas a negative relationship was obtained for pre-test and post-test VAS scores (*r =* −0.307, *p* = 0.054).

Regarding the correlation between socio-emotional competencies, a positive and significant association was observed when comparing the overall workload scores with the subscales related to the mental (*r =* 0.488, *p* = 0.001), temporal demand (*r =* 0.395, *p* = 0.012) and effort (*r =* 0.428, *p* = 0.006) dimensions. For workload dimensions, a negative and moderate association was found between mental demands and the pre-test (*r =* −0.448, *p* = 0.004) and post-test (*r =* −0.317, *p* = 0.047) scores of the STAI scale. On the other hand, a positive relationship was obtained for mental demands with regard to the pre-test VAS score (*r =* 0.423, *p* = 0.006). Physical demand was also significantly associated with pre-test STAI scores (*r =* 0.344, *p* = 0.030), while effort was negatively correlated with the pre-simulation diastolic blood pressure (*r =* −0.335, *p* = 0.034) and performance (*r =* 0.448, *p* = 0.004). At the same time, performance was significantly associated with frustration level (*r =* −0.361, *p* = 0.002), whereas the latter was correlated with blood oxygen saturation post-test (*r =* −0.473, *p* = 0.002).

With respect to the SIMS demotivation dimension, a significant association was demonstrated with external regulation (*r =* 0.347, *p* = 0.028) and performance workload dimension (*r =* 0.506, *p* = 0.001). The self-efficacy scores also presented statistically significant correlations with demotivation (*r =* 0.325, *p* = 0.041) and pre-test STAI values (*r =* −0.445, *p* = 0.004). Likewise, self-efficacy was negatively associated with post-test VAS (*r =* −0.467, *p* = 0.002) scores and frustration level (*r =* −0.406, *p* = 0.009).

Lastly, multiple regression analysis showed a relationship between CPR global effectiveness and (i) post-test blood oxygen saturation, (ii) heart rate and (iii) workload (physical demand) (R^2^ = 0.490; F (3, 39) = 8.305; *p* < 0.0001; *d =* 1.663), thus explaining 49% of the variance of CPR global effectiveness (Table 3). Another regression model displayed the association of CPR effectiveness with self-efficacy and workload (physical demand) (R^2^ = 0.208; F (2, 39) = 4.870; *p* < 0.013; *d =* 1.986). Significant regression equations were also obtained using self-efficacy as a dependent variable and pre-STAI, post-VAS and CPR effectiveness as predictors (R^2^ = 0.431; F (3, 39) = 5.247; *p* < 0.0001; *d =* 1.410) (Table 4). Similarly, mental demands were related with pretest STAI and VAS (R^2^ = 0.358; F (2, 39) = 10.311; *p* < 0.0001; *d =* 1.846) whereas frustration levels were associated with blood oxygen saturation and STAI pre-test (R^2^ = 0.326; F (2, 39) = 8.945; *p* < 0.001; *d* = 2.102).

## 4. Discussion

The purpose of this study was to explore the psychophysiological response and socio-emotional competencies of nursing students during a clinical simulation practice. The effect of stress and anxiety levels, as well as the variation of vital signs and socio-emotional skills, were examined before and after the completion of the simulation. The effectiveness of the learning process was also evaluated according to the physiological parameters and the scores obtained using both psychological and socio-emotional validated questionnaires. Although many previous studies have analyzed the influence of psychological and physiological indicators on simulation performance, this is the first study to report the complementary effect of psychophysiological stress and socio-emotional competencies on training outcomes.

Primary findings revealed a statistically significant increase in heart rate and blood pressure, either systolic and diastolic, regarding baseline levels [10,26]. The cardiovascular response resulting from the overstimulation of the sympathetic nervous system was associated with the activation of the fight-or-flight response [10,24]. The same physiological arousal has been previously reported in different simulation settings involving sport, military or clinical training [10,25,26,40,41]. These studies suggested that the physiological stress provoked by simulation environments leads to a subjective response and a psychological effect on participants [11,26]. In this line, psychological stress measured by VAS and STAI scales was comparable to previous works [26,28,31,37]. Most of the participants showed low stress and anxiety levels, which resulted in good clinical performance in the simulated scenario. The lack of anticipatory stress may be explained by the participants’ confidence in their coping skills to manage a demanding task. Conversely, the anxiety perceived by nursing students increased after the simulation practice. This pattern could be attributed to the perception of the simulation setting as stressful and was consistent with other investigations where higher stress and anxiety scores were obtained in the post-simulation phase [42,43,44]. Furthermore, anxiety levels at the end of the simulation were higher in participants who were more anxious in the pre-simulation stage [44]. As expected, the cardiovascular response explained the positive and significant association between heart rate and blood pressure as well as the negative relationship of blood oxygen saturation to the rest of the variables. Similarly, most of the physiological markers showed a significant correlation with the anxiety experienced by participants post-simulation. This coincides with previous findings in which the stress overall response was related to both physiological and psychological measures [11]. Therefore, the quantification of both components seemed to be crucial to providing a comprehensive view of the subjective stress response.

The participants of this study exhibited high scores in the NASA TLX workload questionnaire, thus indicating a better capacity to perform either cognitive or physical tasks [31]. In this sense, the workload scores were congruent with the elevated effectiveness obtained for CPR simulation. Particularly, both total cognitive load and mental and physical demand dimensions showed a negative association with the global performance of the clinical practice. These results coincided with previously published data in which clinical performance had been negatively correlated with the cognitive load of either health care providers or nursing and medical students [45,46,47,48]. Although a significant association was not found, this finding supports the cognitive load theory about the fatigue caused by a mentally and physically demanding task. In addition, differences between undergraduate and postgraduate students confirmed the effect of the participants’ experience on the decrease in working memory demands thereby enabling a better performance [17]. Thus, undergraduate students with lower domain-specific experience achieved significantly lower performance scores and a higher cognitive load. Regarding the influence of psychophysiological stress and anxiety on the cognitive load, the activation of the sympathetic nervous system had a negative effect on memory and decision making, while higher anxiety levels produced greater mental processing demands [17,25]. From this perspective, the mental demands of participants were negative and moderately associated with both pre- and post-STAI scores. Although anxiety did not lessen the quality of simulation performance, this finding was in line with the hypothesis that suggests that the impairment of cognitive processing is more influenced by anxiety than performance [49]. On the other hand, the relationship between the effort dimension and blood oxygen saturation confirmed the effect of sympathetic activity on the performance of a physically fatiguing task.

The SIMS scale was used to analyze the motivation of participants before the simulation practice. Nursing students obtained medium-to-high scores in the majority of dimensions, which were in accordance with the effectiveness attained during the simulation. Specifically, intrinsic motivation, external regulation and demotivation were significantly correlated with technical skills related to CPR performance such as ventilation or continuous compression time (data not shown). The low stress and anxiety levels reported by participants in this study are consistent with the achievement of higher motivation scores and optimal clinical performances. These results are supported by other studies in which motivation to succeed was linked to reduced stress and anxiety levels [31,50]. Likewise, a significantly negative correlation was found between heart rate in the post-simulation phase and external regulation. Since external regulation, namely the decision to do something “because of the good the activity will do” can be considered as one type of extrinsic motivation, the negative impact on heart rate may be associated with a greater physiological response [21,25].

The analysis of self-efficacy among nursing students yielded interesting results concerning the relationship with either psychophysiological indicators or socio-emotional skills. First, a significant association was observed between self-efficacy scores and simulation performance. Similar results have been reported in studies when the self-efficacy of nursing students concerning the performance of either academic or simulation activities [31,50,51] was evaluated. The confidence of participants in completing a complex task reflects their ability to manage emotions while controlling stress in a threatening situation. In this vein, significant associations were found between self-efficacy and stress and anxiety scores. Low levels of stress led to a high perception of self-efficacy and greater clinical performance. On the other hand, diminished levels of anxiety before the simulation were linked to a higher perceived self-efficacy [52]. Furthermore, the relationship with emotional competencies was revealed by a significant and negative association with demotivation and a moderate positive association with the workload dimension that measured performance. These results were congruent with the high self-efficacy reported by students. Owing to the relation with clinical performance, self-efficacy appears to be a valuable tool in evaluating the development of competencies [3].

Several studies demonstrated that psychophysiological stress negatively affects clinical performance, while emotional control plays a key role in critical situations. To confirm this assumption, the influence of these variables on CPR effectiveness was performed via multiple-regression analysis. Although the link between clinical performance and all the parameters studied was not proven, a consistent pattern was found from the measurement of physiological and socio-emotional records. The analysis of multiple regression models showed that heart rate, blood oxygen saturation, physical demand and self-efficacy can be used as predictors of training outcomes. Furthermore, a significant regression equation was obtained when considering self-efficacy as a dependent variable, showing the association with VAS post-test scores, pre-test STAI scores and the global effectiveness of CPR.

Despite these promising findings, this study presents several limitations that should be highlighted. First, the study was limited by the small sample size and the selection of participants by convenience sampling. Additionally, psychological and socio-emotional variables were self-reported by participants, thus resulting in a potential bias due to the desire to provide a positive self-image. The time required to complete the questionnaires was also a possible limitation. Lastly, since stress levels may vary according to the simulation setting, the study could be replicated in other clinical environments. Therefore, results may not be generalized to real-life clinical scenarios. Likewise, the sociodemographic characteristics of participants are not representative of a registered nurse cohort.

## 5. Perspectives on Nursing Education and Clinical Practice

This study offers a useful approach for predicting the quality of clinical simulated practices by taking advantage of both psychophysiological and socio-emotional indicators. In particular, our results suggest that (i) the participation in the simulation practice induced a psychophysiological response in nursing students; (ii) socio-emotional competencies such as workload, motivation and self-efficacy are closely related to the anxiety and stress levels of participants; and (iii) the effectiveness of a simulation performance is associated with the physiological stress, self-efficacy and workload perceived by participants.

These findings have implications for nursing education and clinical practice. First, the importance of controlling psychophysiological stressors is crucial for dealing with stressful learning experiences. Thus, educators should expose learners to simulation scenarios tailored to their clinical experience and instruction level. On the other hand, nurse educators should reinforce the development of socio-emotional competencies to improve the acquisition of specific skills. Student proficiency in performing a simulation event relies upon the congruence between cognitive demands and the domain-specific experience of participants. Therefore, the complexity of the simulation practice needs to be adjusted to the experience and socio-emotional skills of the learner to cope with stress in clinical situations. Mastery over emotions may lead not only to better simulation outcomes but also to optimal professional performance and improved health-care quality.

## 6. Conclusions

The appraisal of psychological, physiological and socio-emotional indicators to determine simulation outcomes is restricted to a limited number of studies. This work investigated the complementary effect of psychophysiological stress and socio-emotional skills on clinical performance. The effectiveness of a simulated practice based on a cardiopulmonary resuscitation scenario was associated with physiological parameters such as heart rate and blood oxygen saturation. The clinical performance also exhibited a consistent trend with perceived self-efficacy and cognitive load dimensions. The impact of self-reported stress and anxiety showed good correlations with physiological changes in blood pressure, self-rated motivation, self-efficacy and workload mental demand. These findings suggest that psychophysiological stress has negative consequences on clinical performance. Similarly, the assessment of emotional skills provides an understanding of the performance of students during clinical simulated practice. Therefore, using socio-emotional and psychophysiological markers simultaneously may contribute to preventing the influence of undesirable stressors while predicting the effect on training outcomes. More research is needed to enable the control of the learning environment by evaluating the effect of emotional, physiological and psychological factors on optimal simulation performances.

## Figures and Tables

**Table 1:** Physiological and psychological variables at pre-test and post-test moments.

Variables	Moment		Statistic	*p*-Value
	Pre-Test	Post-Test	*t*-Paired	
Systolic blood pressure	109.62 ± 15.428	117.82 ± 10,818	−4.962	0.0001 **
Diastolic blood pressure	70.55 ± 8.765	71.43 ± 8.461	−0.792	0.433
Heart rate	73.83 ± 12.302	86.10 ± 18.267	−5.604	0.0001 **
Oxygen saturation	97.95 ± 1.037	97.95 ± 0.783	0.0001	1.000
STAI scores	23.50 ± 4.90	24.40 ± 4.97	−1.099	0.278
VAS scores	3.00 ± 1.88	3.22 ± 2.39	−0.691	0.494

Mean values ± SD. Comparisons made using paired Student’s *t* test. ** *p* < 0.01.

**Table 2:** Descriptive statistics of self-reported socio-emotional competencies (workload, motivation and self-efficacy) using the NASA TLX workload, the SIMS motivation scale, the self-efficacy CPR scale and their corresponding subscales.

Instrument	Scores	Statistic	*p*-Value
		*t*-Test	
NASA TLX workload			
NASA overall	64.675 ± 14.51582	28.179	0.0001 **
Mental demand (M)	177 ± 131.172	8.534	0.0001 **
Physical demand (F)	104.88 ± 102.504	6.471	0.0001 **
Temporary demand (T)	157.88 ± 113.006	8.836	0.0001 **
Effort (E)	279.63 ± 117.17	15.094	0.0001 **
Performance (P)	139.88 ± 118.124	7.489	0.0001 **
Frustration (FR)	110.88 ± 124.21	5.646	0.0001 **
**SIMS Motivation**			
Intrinsic Motivation	21.58 ± 4.314	31.631	0.0001 **
Regulation identified	23.23 ± 4.086	35.953	0.0001 **
External regulation	12.48 ± 5.809	13.582	0.0001 **
Demotivation	5.65 ± 3.431	10.415	0.0001 **
**Self-efficacy CPR**			
	34.08 ± 6.375	33.808	0.0001 **

Mean values ± SD. Comparisons made using Student’s *t* test. ** *p* < 0.01. Scales are represented in bold text.

**Table 3:** Multiple linear regression analysis to model the relationship between global CPR effectiveness scores and heart rate, blood oxygen saturation and physical demand workload.

Dependent Variable: CPR Effectiveness	Unstandardized Coefficients		Standardized Coefficients Beta	Significance
	B	Standard Error		
Constant	−1050.400	289.634		
Oxygen saturation	11.287	2.952	0.490	0.001
Heart rate	−0.057	0.023	−0.322	0.017
Physical demand	0.290	0.127	0.294	0.028

**Table 4:** Multiple linear regression analysis to model the relationship between self-efficacy scores and stress (VAS pre-test) and anxiety levels (STAI post-test) and CPR effectiveness.

Dependent Variable: Self-Efficacy	Unstandardized Coefficients		Standardized Coefficients Beta	Significance
	B	Standard Error		
Constant	16.638	6.037		
VAS pre-test	−0.859	0.352	−0.323	0.020
STAI post-test	0.528	0.168	0.406	0.03
CPR effectiveness	0.105	0.046	0.298	0.028

## Data Availability

Data will be available upon reasonable request to corresponding author.
